# Ankle ligament reconstruction‐return to sport after injury scale and return to sports after ankle ligament reconstruction or repair—A systematic review

**DOI:** 10.1002/jeo2.12077

**Published:** 2024-07-02

**Authors:** YuChia Wang, Maximilian Hinz, Wyatt H. Buchalter, Amelia H. Drumm, Emre Eren, C. Thomas Haytmanek, Jonathon D. Backus

**Affiliations:** ^1^ Steadman Philippon Research Institute Vail Colorado USA; ^2^ Department of Sports Orthopaedics Technical University of Munich Munich Germany; ^3^ The Steadman Clinic Vail Colorado USA

**Keywords:** ALR‐RSI, ankle instability, ankle ligament reconstruction, ankle ligament reconstruction‐return to sport after injury scale, systematic review

## Abstract

**Purpose:**

To systematically review existing literature regarding the ankle ligament reconstruction‐return to sport after injury (ALR‐RSI) scale and to assess its correlation with Return to sport and functional outcomes as well as feasibility, reliability and consistency.

**Methods:**

A systematic review of the literature based on the Preferred Reporting Items for Systematic Reviews and Meta Analyses (PRISMA) was conducted using PubMed, Embase and Cochrane Library. Studies that evaluated psychological readiness to return to sport after ankle ligament reconstruction or repair for the treatment of chronic lateral ankle instability using the ALR‐RSI scale were included. The results from each study were pooled, and weighted means and overall rates were calculated.

**Results:**

In total, 157 patients (53.2% male, mean age: 34.2 years) from three articles were included. Overall, 85.0% of patients reported successful return to sport, but only 48.9% of patients returned to the preoperative sporting level. All studies reported a significant difference in psychological scores between patients who returned to sport and those who did not. Pooled mean patient‐reported outcome measures, reported as the American Orthopaedic Foot and Ankle Society Ankle‐Hindfoot (AOFAS, three studies) Score and Karlsson‐Peterson Score (three studies), were 82.7 (range: 29–100) and 81.7 (range: 25–100), respectively. The ALR‐RSI scale demonstrated strong correlations with the AOFAS Score and Karlsson‐Peterson Score.

**Conclusion:**

Patients who returned to sport after ankle ligament reconstruction or repair exhibited higher psychological readiness compared to those who did not. The ALR‐RSI scale showed strong correlations with ankle function. Evaluation of psychological readiness using the ALR‐RSI scale may provide an additional tool in the assessment of patients who underwent ankle ligament reconstruction or repair.

**Level of Evidence:**

Level III, systematic review.

**Abbreviations**
ACL: anterior cruciate ligament
ALR‐RSI: ankle ligament reconstruction‐return to sport after injury
AOFAS: American Orthopaedic Foot and Ankle Society
CALI: chronic ankle ligament instability
FAI: femoroacetabular impingement
Hip‐RSI: hip‐return to sport after injury
MINORS: Methodological Index for Non‐Randomized Studies
MPFL‐RSI: medial patellofemoral ligament‐return to sport after injury
PRIMSA: Preferred Reporting Items for Systematic Reviews and Meta Analyses
RTS: return to sport
SIRSI: shoulder instability‐return to sport after injury

## INTRODUCTION

Inversion ankle trauma and ankle sprains are among the most common sports‐related injuries, accounting for 4%–7% of all emergency department chief complaints, representing an economic burden estimated at about $2 billion per year in the United States of America alone [13, 35]. While a conservative approach with protective bracing and physical therapy may be a traditional first‐line treatment [41], up to 20% of patients may go on to develop chronic lateral ankle instability (CLAI) [9, 12, 19].

Several surgical treatment options have been described to restore ankle stability [31, 40]. Repair of ankle ligaments with a modified Broström–Gould procedure, consisting of lateral ligament imbrication with direct suture repair and transfer of the extensor retinaculum, remains the current gold standard for surgical management of CLAI [29, 40]. However, it has been reported that 25% of patients are unable to return to sport [7, 12, 22, 23]. This is concerning as the ability to return to prior sporting activities is one of the main expectations of patients prior to undergoing surgery [20, 21, 27, 33].

Traditionally, return to sport has been reported in metrics that do not take into account psychological factors such as an individual's motivation, self‐confidence in performance, and fear. Physical impairments alone are, however, not sufficient to explain the lower‐than‐expected return to sport rates following sport‐related injuries, thereby highlighting the potential role that psychological factors have in the return to sport process [4, 28]. As such, measures that assess psychological factors have been developed to properly assess each patient's ability to return to sport after surgical intervention. Webster et al. designed the ACL‐return to sport after injury (ACL‐RSI) scale to assess patients' psychological readiness to resume sports after anterior cruciate ligament (ACL) reconstruction [43]. Since the validation of the ACL‐RSI Scale, numerous psychological assessment scales for return to sport have been extrapolated to other pathologies and associated surgical interventions, including the shoulder instability‐return to sport after injury (SIRSI) for shoulder instability [10], the hip‐return to sport after injury scale (Hip‐RSI) for hip arthroscopy with femoroacetabular impingement syndrome [45], the medial patellofemoral ligament‐return to sport after injury scale (MPFL‐RSI) for medial patellofemoral ligament reconstruction [14], and the ankle ligament reconstruction‐return to sport after injury (ALR‐RSI) for ankle ligament reconstruction or repair [1, 26, 27, 34]. Few studies have been published on the ALR‐RSI scale and its relation to return to sport, but there exists a paucity of literature reporting a pooled analysis of these studies [1, 26, 27, 34].

The purpose of the present study was to systematically review the existing literature regarding the ALR‐RSI Scale and its importance in the context of return to sport after ankle ligament reconstruction or repair. It was hypothesized that the ALR‐RSI scale would be an adequate measure in assessing patients' ability and readiness to return to sport after ankle ligament reconstruction or repair.

## MATERIALS AND METHODS

### Search strategy

A systematic review was conducted in accordance with the 2020 Preferred Reporting Items for Systematic Reviews and Meta Analyses (PRISMA) guidelines [25] and registered on the PROSPERO International prospective register of systematic reviews (ID: CRD42023480735). A literature search was conducted to identify studies that reported on the ALR‐RSI scale in the context of ankle ligament reconstruction or repair for the treatment of CLAI by querying PubMed, Embase and the Cochrane Library from inception through 27 October 2023. The following search terms combined with Boolean operators: ‘ankle’, ‘return to sport’, ‘psychological readiness’, ‘return to sport after injury’ and ‘RSI’.

### Eligibility criteria

Inclusion criteria consisted of peer‐reviewed studies available in English that reported the ALR‐RSI scale following ankle ligament reconstruction or repair for the treatment of CLAI in at least five patients. Studies that did not assess the ALR‐RSI scale following ankle ligament reconstruction or repair and those that reported the ALR‐RSI scale, but in a different context, were excluded.

Two authors (YuChia Wang and Amelia H. Drumm) independently conducted an initial title and abstract screening followed by a full‐text screen to determine whether studies met the inclusion criteria. A third independent author (Jonathon D. Backus) was available to discuss and resolve any disagreement, but no disagreements were encountered. Reference lists from the included studies were cross referenced to ensure that all relevant articles meeting the inclusion criteria would be included in this systematic review.

### Data extraction

Data were extracted from the included studies and entered into a Microsoft® Excel spreadsheet (Version 16.77.1, Microsoft). Data extracted from each study included study characteristics, patient and surgery‐related data, functional outcomes, as well as ALR‐RSI scale reliability, feasibility and internal consistency.

### Data and statistical analyses

Weighted mean of postoperative functional outcomes, ALR‐RSI scale correlation to the reported functional outcome measures, and the ALR‐RSI scale‐related reliability, feasibility and consistency evaluations were pooled.

### Risk‐of‐bias assessment

A methodological quality assessment was performed on all included studies by two investigators (YuChia Wang and Wyatt H. Buchalter) independently using the Methodological Index for Non‐Randomized Studies (MINORS) criteria to minimize bias. A third investigator was available to resolve any disagreements (Jonathon D. Backus).

## RESULTS

**Figure 1:**
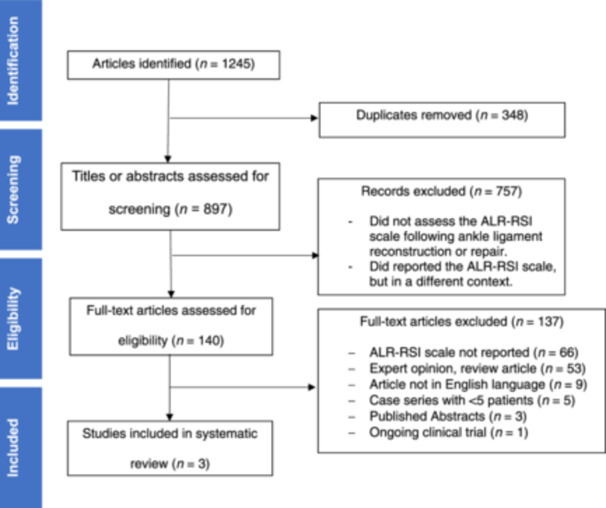
PRISMA flow diagram. ALR‐RSI, ankle ligament reconstruction‐return to sport after injury. PRISMA, Preferred Reporting Items for Systematic Review and Meta‐Analyses.

The initial literature search yielded a total of 1245 articles. Following the removal of 348 duplicates, 897 articles were screened for eligibility based on a title and abstract review, through which 757 articles were excluded. One hundred and forty articles were selected for full‐text review, of which three were included in this review (Figure 1). Of the 137 articles that were excluded, 66 did not report assessing the ALR‐RSI scale, 53 articles were expert opinions or review articles, nine articles were not in English language, five articles were case series with less than five patients, three articles were abstracts without an associated full text, and one article was an ongoing clinical trial.

### Study characteristics and risk‐of‐bias assessment

Of the included studies, one study was level two evidence, and two studies were level three evidence. The included studies were published in the following journals: *Knee Surgery, Sports Traumatology, Arthroscopy* (*n* = 2) [27, 34] and the *Journal of Experimental Orthopaedics* (*n* = 1) [1]. The mean overall MINORS score was 11 (range: 10–12) for level three studies and 9.5 (range: 8–11) for the level two study.

### Patient characteristics

In total, 158 patients (160 ankles) were included (53.2% male). Two studies reported the mean age at time of surgery (34.2 years, range: 18–63 years) [1, 27]. All three included studies reported on preoperative sporting activity levels, with most participating at an amateur level (96.2%), and 3.82% (6/157) of patients participating in sporting activity at a professional level prior to surgical intervention [1, 27, 34]. The included patients underwent an arthroscopic lateral ankle ligament reconstruction [1, 34], arthroscopic ankle ligament repair [1], or arthroscopic Modified Broström‐Gould (Table 1) [27]. Prior nonoperative treatment was not reported by any study. Follow‐up was reported by two studies (mean: 2.8 years) and ranged from 2.0 to 3.7 years [27, 34]. Patient characteristics are summarized in Table 1.

**Table 1:** Patient characteristics.^a^

Lead author (year)	Patients (ankles) followed up, *N*	Surgery performed	Sex, %	Age at surgery, years (range)	Follow‐up time, years (range)	Preoperative sporting level
Sigonney (2020) [34]	57 (59)	Arthroscopic anatomical lateral ankle ligament reconstruction	52.6% (30/57) male 47.4% (27/57) female	NR	3.0 years (2.5–3.7)	44.1% (26/59) Regular leisure level 6.77% (4/59) Casual leisure level 49.2% (29/59) Competition level
Pioger (2022) [27]	71	Arthroscopic modified Broström‐Gould procedures	50.7% (36/71) male 49.3% (35/71) female	34.1 (18–63)	2.6 years (2.0–3.7)	46.5% (33/71) Regular leisure level 15.5% (11/71) Casual leisure level 35.2% (25/71) Competition level 2.81% (2/71) Professional level
Ajaka (2022) [1]	29	Arthroscopic anatomical lateral ankle ligament reconstruction or repair	60.0% (18/30) male 40.0% (12/30) female	34.6	NR	13.8% (4/29) Professional level 86.2% (25/29) Amateur level

Abbreviation: NR, not reported.

^a^ Continuous data are presented as mean (range).

### Return to sport

Return to sport rates were reported by all three studies [1, 27, 34] with a pooled RTS rate of 82.7% (range: 77.5%–96.7%). Of the included patients who successfully returned to sporting activities, 48.9% reported return to previous level of activity, 29.6% reported return to an inferior level, and 21.5% reported performing a different sport postoperatively. Of note, one included study reported the mean time to return to sport after surgery (5.6 months) [1].

### Patient‐reported outcome measures

Foot and ankle functional outcome measures were also reported by all studies. All three included studies reported the American Orthopaedic Foot and Ankle Society (AOFAS) Ankle‐Hindfoot Scale and the Karlsson‐Peterson scale. The AOFAS Score entails a scale from 0 to 100 that evaluates pain, function and alignment of the ankle with lower scores indicating higher degrees of pain and impairment [32]. The Karlsson‐Peterson Score assesses ankle joint stability and function on a 0–100 scale with higher scores indicating higher degrees of ankle stability and function ankle as well as higher ability to perform activities of daily living, such as running, and stair climbing [15, 16]. The pooled mean reported AOFAS Scale was 82.7 (range: 29–100) and the pooled mean reported Karlsson‐Peterson Score was 81.7 (range: 25–100) [1, 27, 34].

### ALR‐RSI scale

All studies evaluated the ALR‐RSI scale exclusively postoperatively. Two studies reported when psychological readiness via the ALR‐RSI scale was evaluated (2.6 years [range: 2.0–3.7 years] [27] and 3.0 years [2.5–3.7 years]) [34]. The third study did not report follow‐up time [1]. Across all three included studies, significant postoperative differences regarding the ALR‐RSI scale were reported between athletes who successfully returned to sporting activities versus those who did not return to sporting activities. The pooled mean of ALR‐RSI across all three studies, regardless of return to sport status, was 65.1 (range: 10–100). The weighted mean of ALR‐RSI for patients who successfully returned to sport at follow‐up was 70.9 whereas the weighted mean was 45.1 in those who did not return to sport, with reported *p*‐values ranging from <0.01 to 0.02. Differences regarding the ALR‐RSI scale in patients that did versus those that did not RTS are summarized in Table 2.

**Table 2:** ALR‐RSI scale.^a^

Lead author (year)	ALR‐RSI scale with successful return to sport	ALR‐RSI scale without successful return to sport	*p*‐value
Sigonney (2020) [34]	68.8 (56.6–86.5)	45.0 (31.3–55.8)	0.02
Pioger (2022) [27]	61.9 (43.8–79.6)	43.4 (25.0–55.6)	0.01
Ajaka (2022) [1]	91.6 (NR)	72.7 (NR)	<0.01
Pooled mean	70.9 (43.8–86.5)	45.1 (25.0–55.8)	

Abbreviations: ALR‐RSI, ankle ligament reconstruction‐return to sport after injury scale; NR, not reported.

^a^ Continuous data are presented as mean (range).

All three included studies also assessed the internal consistency with a Cronbach *α* coefficient of *α* ≥ 0.90 considered excellent by all included studies. The percentage of missing responses, the ceiling and floor effects were utilized by all studies to evaluate the feasibility of the ALR‐RSI scale. More specifically, the presence of a floor or ceiling effect of more than 15% was considered problematic with the questionnaire validity in a previous study on evaluating quality criteria for measurement properties of health status questionnaires [38]. In order to assess the reliability of the ALR‐RSI scale, a test‐retest was conducted by all three studies with an interval ranging from 3 days to 2 weeks. The *ρ* intraclass correlation coefficient (ICC) was then used to evaluate the reliability. Table 3 provides an overview of the reported validation measures, indicating a scoring system that is feasible, reliable and consistent.

**Table 3:** ALR‐RSI scale validation measures.^a^

Lead author (year)	Feasibility (%)		Reliability (Median, range)			Internal consistency Cronbach *α*‐coefficient
	Floor	Ceiling	First	Second	*p*‐Value	
Sigonney (2020) [34]	0–1.7	6.7–33.8	66.3 (45.6–85.8)	57.1 (38.1–79.0)	0.92 (0.86–0.96)	0.96
Pioger (2022) [27]	0–1.7	4.2–35.2	58.3 (41.2–77.5)	59.2 (37.5–80.4)	0.93 (0.86–0.96)	0.95
Ajaka (2022) [1]	0–3.3	3.3–9.7	83.1 (NR)	83.8 (NR)	0.97 (0.94–0.99)	0.94

Abbreviations: ALR‐RSI, ankle ligament reconstruction‐return to sport after injury; NR, not reported.

^a^ Data are reported in percentage (feasibility), and median (range).

### Correlation with functional outcomes

The ALR‐RSI scale demonstrated a correlation of *r* > 0.5 with ankle function (AOFAS and Karlsson‐Peterson Scores), which were interpreted as ‘strong’ by all three studies [1, 27, 34]. More specifically, the mean correlation of ALR‐RSI to AOFAS Score reported by included studies ranged from *r* = 0.72 to 0.90. The mean correlation of ALR‐RSI to Karlsson‐Peterson Score ranged from *r* = 0.69 to 0.85.

## DISCUSSION

This systematic review examined three studies that evaluated the ALR‐RSI scale and its utility for evaluating successful return to sport in the context of ankle lateral ligament reconstruction or repair. The most significant finding of this systematic review was that patients who returned to sport scored significantly higher on the ALR‐RSI scale compared to those who did not. This specific finding was essential in demonstrating the association between psychological readiness and return to sport, indicating that the psychological aspect of returning to sport may be as important as physical readiness. All studies reported mean postoperative AOFAS and Karlsson‐Peterson Scores greater than 80 following ankle ligament reconstruction or repair, indicating good to excellent overall functional outcomes. All included studies reported high return to sport rates with a mean value of 85.0% and strong correlations between the ALR‐RSI scale and ankle function. The included studies also reported that the ALR‐RSI scale was feasible, reliable and internally consistent, suggesting the scale could subsequently be incorporated into the rehabilitation progress of patients aiming to return to sport. Ajaka et al. reported that on average patients required 5.6 months to return to sport, but did not explicitly report when the ALR‐RSI scale was utilized [1]. It may be speculated that the authors implemented the ALR‐RSI scale at this time as the patients returned to their respective sports. It should be noted, however, that the average follow‐up time from the reported studies were 2.8 years. Webster et al. showed that of the patients who underwent either primary or revision ACL reconstruction, only 24% who expected to return to preinjury level of sport had actually returned [42]. Ardern et al. found that only two of every five athletes were able to participate at preinjury level of sport at 2 years after ACL reconstruction. Together suggesting that it may be difficult to return to prior level beyond 1 year [5].

Although the current study examined psychological metrics after patients returned to sporting activities, psychological factors may change throughout a patient's postoperative recovery journey. As demonstrated by Xiao et al. [46] in their meta‐analysis on the ACL‐RSI scale, the RSI scores significantly increased over the course of 3, 6 and 12 months postoperatively, indicating that patients experienced fewer negative emotions associated with their injuries and felt more confident about returning to sport the further along they progressed in rehabilitation. Sadequ et al. [30] and Toale et al. [39] also demonstrated that the ACL‐RSI scores improved significantly up to 2 years postoperatively. As such, not only should psychological factors be evaluated in postoperative patients, but these factors should also be evaluated at multiple time points throughout the rehabilitation process in order to identify those who may be at risk for not achieving successful return to sport.

Because lower ALR‐RSI scores were associated with lower return to sport rates, psychological readiness screening may be beneficial. This aligns with the findings not only from the present review, but also with the evaluation of the RSI scale utilized in different pathologies, such as in shoulder instability [24, 31], in femoroacetabular impingement [45], and patellofemoral instability [14]. Regardless of the pathology or surgical interventions involved, the RSI scale demonstrated a significant association to successful return to sport between different pathologies and procedures across multiple joints.

According to Wikstrom et al. [44] and Tassignon et al., [37] no consensus criteria currently exist to inform return to sport decisions for patients with lateral ankle ligament injuries. It was suggested that the decision to resume sporting activities may base upon functional testing that includes sport‐specific movement, static balance, range of motion and strength in conjunction with patient‐reported outcome measures. Because hopping and balancing tests evaluate different aspect of ankle function, it may be relevant to combine these during the return to sport decision‐making process. Ko et al. [18] suggested that a combined functional performance tests rather than a single test improves the clinical value of testing. Nevertheless, Clanton et al. [6] suggested that the psychological aspect must be taken into consideration throughout the return to sport decision‐making process, as 5%–19% of athletes experienced psychological distress following injury to levels comparable with individuals' receiving treatments for mental health illness [11]. Athletes who demonstrated apprehension, fear, or anxiety are at a much greater risk of reinjury with an often‐associated deleterious impact on athletic performance [3, 8, 36, 41, 43, 44]. Therefore, psychological readiness may be as important as ankle function when determining return to sporting activities in the rehabilitation process.

Several limitations should be noted for this review. Due to the relatively novel nature of the ALR‐RSI scale as well as the strict inclusion and exclusion criteria, only three studies were considered eligible for inclusion. While all included studies in the present review also included patient‐reported outcome measures that assessed ankle function, they did not report preoperative records of these measures, consequently not allowing for preoperative prediction of postoperative return to sport. The limited number of existing literature and data available makes complete analysis difficult, the lack of standardize ALR‐RSI implementation protocol in clinical practice presents further challenges. If the ALR‐RSI scale were to be implemented in clinical practice, a questionnaire should be given to patients when returned to sport at any level, then once more at an additional follow‐up in order to properly assess whether patients are indeed psychologically ready to return to sporting activities.

Furthermore, the AOFAS Ankle‐Hindfoot Score is not considered reliable or valid and its use is discouraged by the AOFAS currently due to limited evidence supporting its clinical efficacy [2, 17]. The optimal level of psychological readiness that allows for return to sporting activities without being at increased risk for further or repeat injury is also difficult to assess without preoperative measurements for comparisons [46, 47]. Additionally, the lack of range of motion, pain, or postoperative complications reporting made it unfeasible to determine specifically how an individual's psychological readiness was influenced and what factors postoperative recovery played in patients' willingness and readiness to return to sport participation. Lastly, return to sport was probably dependent on several factors beyond psychological readiness and should therefore be considered in conjunction with objective return‐to‐sport measurements such as clinical examinations on postoperative ankle stability and functional testing.

## CONCLUSION

The ALR‐RSI scale is a valid and effective measurement for assessing patients' readiness to return to sports after undergoing ankle ligament reconstruction or repair for CLAI. It successfully discriminated between patients who returned to sports successfully and those who did not. It may also assist in determining which patient is potentially at risk for not achieving successful return to sport. The further implementation of the ALR‐RSI scale may allow clinicians to counsel patients with an additional psychological emphasis throughout the postoperative rehabilitation process.

## AUTHOR CONTRIBUTIONS

YuChia Wang and Wyatt H. Buchalter performed data extraction and wrote the manuscript. YuChia Wang and Maximilian Hinz conceptualized the review. YuChia Wang, Wyatt H. Buchalter and Amelia H. Drumm searched the literature. Maximilian Hinz, Emre Eren, C. Thomas Haytmanek and Jonathon D. Backus performed critical revision of the manuscript. C. Thomas Haytmanek and Jonathon D. Backus supervised the manuscript. All authors read and approved the final manuscript.

## CONFLICT OF INTEREST STATEMENT

C. Thomas Haytmanek has received research support from Stryker, consulting fees from Arthrex, speaking fees from Gemini Mountain Medical, Steelhead Surgical, hospitality payments from Integra, Bioventus, Exactech and Stryker, and support for education from Wright Medical Technology. Jonathon D. Backus has received royalties and consulting fees from Medline Unite, holds stock options for Marrow Access Technologies, and is a board and committee member in AOFAS. The remaining authors declare no conflict of interest.

## ETHICS STATEMENT

Not applicable.

## Data Availability

Data sharing is not applicable to this article as no datasets were generated or analysed during the current study.
